# Mortality in psychotic depression: 18-year follow-up study

**DOI:** 10.1192/bjp.2022.140

**Published:** 2023-01

**Authors:** Tapio Paljärvi, Jari Tiihonen, Markku Lähteenvuo, Antti Tanskanen, Seena Fazel, Heidi Taipale

**Affiliations:** Department of Forensic Psychiatry, University of Eastern Finland, Niuvanniemi Hospital, Finland; and Department of Psychiatry, Oxford University, Warneford Hospital, UK; Department of Forensic Psychiatry, University of Eastern Finland, Niuvanniemi Hospital, Finland; and Department of Clinical Neuroscience, Karolinska Institutet, Sweden; Department of Forensic Psychiatry, University of Eastern Finland, Niuvanniemi Hospital, Finland; Department of Psychiatry, Oxford University, Warneford Hospital, UK; and Oxford Health NHS Foundation Trust, Warneford Hospital, UK; Department of Forensic Psychiatry, University of Eastern Finland, Niuvanniemi Hospital, Finland; Department of Clinical Neuroscience, Karolinska Institutet, Sweden; and School of Pharmacy, University of Eastern Finland, Finland

**Keywords:** Cohort study, depression, epidemiology, psychotic disorders, mortality

## Abstract

**Background:**

Evidence on the role of co-occurring psychiatric disorders in mortality associated with psychotic depression is limited.

**Aims:**

To estimate the risk of cause-specific mortality in psychotic depression compared with severe non-psychotic depression while controlling for comorbid psychiatric disorders.

**Method:**

This cohort study used routine data from nationwide health registers in Finland. Eligible participants had their first diagnosis for psychotic depression or for severe non-psychotic depression between the years 2000 and 2018, had no pre-existing diagnoses for schizophrenia spectrum disorders or bipolar disorder, and were aged 18–65 years at the index diagnosis. Causes of death were defined by ICD-10 codes. The follow-up time was up to 18 years.

**Results:**

We included 19 064 individuals with incident psychotic depression and 90 877 individuals with incident non-psychotic depression. Half (1199/2188) of the deaths in those with psychotic depression occurred within 5 years from the index diagnosis and the highest relative risk was during the first year after the diagnosis. Compared with individuals with non-psychotic depression, those with psychotic depression had a higher risk of all-cause mortality (adjusted hazard ratio, aHR = 1.59, 95% CI 1.48–1.70), suicides (aHR = 2.36, 95% CI 2.11–2.64) and fatal accidents (aHR 1.63, 95% CI 1.26–2.10) during the subsequent 5-year period after the index diagnosis.

**Conclusions:**

Psychotic symptoms markedly added to the mortality risk associated with severe depression after controlling for psychiatric comorbidity. Prompt treatment and enhanced monitoring for psychotic symptoms is warranted in all patients with severe depression to prevent deaths because of suicides and other external causes.

## Background

Psychotic symptoms can complicate mental health disorders independently of severity of the primary condition, including unipolar depression.^1,2^ Psychotic symptoms, such as delusions or hallucinations add to the range of mental health symptoms experienced by the individual and thus add to the total burden of disease. However, the diagnostic threshold for clinically relevant psychotic symptoms is not well established,^2^ and in about one-third of patients with psychotic depression, their psychotic symptoms could be missed clinically.^3^ Psychotic depression is associated with poorer treatment outcomes,^4–6^ higher prevalence of psychiatric comorbidity^7^ and higher mortality than non-psychotic depression,^8,9^ but the disease process of psychotic depression is still relatively unknown.^1,6,10,11^ For example, the evidence is limited in terms of diagnostic stability of psychotic depression, and it has been suggested that psychotic depression could merely be a ‘working diagnosis’ or a misdiagnosis of other psychiatric disorders.^10,12,13^ Very little is known about diagnostic changes, i.e. conversion of psychotic depression to other mental disorders, such as bipolar disorder or schizophrenia after the first episode of psychotic depression.^14–16^

Individuals with psychotic depression have been found to have about twofold higher risk of all-cause mortality compared with individuals with severe non-psychotic depression.^8^ Risk of completed suicide has been reported to be 20–70% higher and risk of suicide attempt about twofold higher in individuals with psychotic depression compared with individuals with non-psychotic depression,^9,17,18^ but the evidence is heterogeneous and mainly based on small sample sizes.^19^ Furthermore, it has been suggested that comorbid psychiatric disorders, such as personality disorder and substance use disorder, could explain a substantial part of the excess mortality in individuals with psychotic depression.^7,10,14–16,20^

## Aims

In this large population-based cohort study of over 19 000 individuals with first-episode psychotic depression followed up for up to 18 years after their index diagnosis, some of the limitations in the evidence base on psychotic depression are addressed. First, the diagnostic stability of psychotic depression is examined by establishing the probability of change of diagnosis after the first episode of psychotic depression, i.e. conversion of psychotic depression to other major groups of psychiatric disorders, including schizoaffective disorder, bipolar disorder and schizophrenia. Second, how the change of diagnosis (i.e. conversion of psychotic depression) is associated with mortality is examined. Third, the relative risk of cause-specific mortality in severe psychotic depression compared with severe non-psychotic depression is established, while adjusting for the effects of history of self-harm, pre-existing personality disorder and substance use disorder, and subsequent conversion to the other major groups of psychiatric disorders.

This study thus adds to the evidence base for the disease process and prognosis of first-episode psychotic depression. Individuals with first-episode severe non-psychotic depression are used as the comparison group in order to control for the severity of the underlying depression.

## Method

We used Finnish nationwide registers to identify individuals diagnosed with depression. The hospital discharge register maintained by the National Institute for Health and Welfare includes information on all in-patient and any type of specialised out-patient care contacts and the registers maintained by the Social Insurance Institution and the Centre for Pensions include information on reimbursed disability-related benefits, including sickness allowances (sick leave ≥14 days) and disability pensions. The hospital discharge register was also used to identify comorbidities. The cause of death register maintained by Statistics Finland was used to identify causes of deaths. We used information from these registers for the years between 1998 and 2018. All diagnoses were identified using the ICD-10, Finnish modification codes (Supplementary Table 1 available at https://doi.org/10.1192/bjp.2022.140). The record linkage was done by the National Institute for Health and Welfare using a unique personal identification number issued to all permanent residents in Finland.

The authors assert that all procedures contributing to this work comply with the ethical standards of the relevant national and institutional committees on human experimentation and with the Helsinki Declaration of 1975, as revised in 2008. Because the study used administrative pseudonymised register data, no institutional review board approval or informed consent were needed. The study design and record linkages were approved by the review boards of the institutions maintaining the registers: The Finnish National Institute for Health and Welfare (635/5.05.00/2019), the Social Insurance Institution of Finland (31/522/2019), Finnish Centre for Pensions (19023) and Statistics Finland (TK-53-569-19). This study follows the Strengthening the Reporting of Observational studies in Epidemiology guidelines.

### Design

Individuals were eligible if they had their first diagnosis (i.e. index diagnosis) of non-psychotic severe depression (ICD-10: F32.2, F33.2) or psychotic severe depression (ICD-10: F32.3, F33.3) between the years 2000 and 2018, had no pre-existing diagnoses for schizophrenia spectrum disorders (ICD-10: F20 – F29) or bipolar disorder (ICD-10: F30, F31), and were aged 18–65 years at the index diagnosis. We focused on the adult population aged up to 65 years at the index diagnosis, because in this age group the likelihood of confounding because of neuropsychiatric and somatic comorbidities underlying first-episode psychosis are likely less prevalent than in older age groups. The ICD-10 classification includes codes for psychotic symptoms only within severe depression, meaning that all patients with psychotic depression were diagnosed to have severe depression.

The cohort for psychotic depression was defined so that when a patient received their first diagnosis for psychotic depression during the study period, they belonged to the psychotic depression cohort regardless of their subsequent psychiatric diagnoses, for example for non-psychotic depression or bipolar disorder. Patients who received a diagnosis for non-psychotic depression, but not for psychotic depression, during the study period belonged to the non-psychotic depression cohort and their first diagnosis for non-psychotic depression was used as the index diagnosis. In other words, patients from the non-psychotic depression cohort did not move to the psychotic depression cohort during the study period.

To control for confounding by diagnostic misclassification and co-occurring severe mental health disorders, we excluded individuals who were diagnosed with schizophrenia spectrum disorders or bipolar disorder within 14 days of the index diagnosis, and individuals who had pre-existing diagnoses for organic mental disorders (e.g. dementia) or intellectual disability (Supplementary Table 1). The 14-day exclusion period for change of diagnosis to schizophrenia spectrum disorders or bipolar disorder was used to exclude patients who very likely had one of these psychiatric disorders at the time of psychotic depression diagnosis, i.e. they had a ‘working diagnosis’ of psychotic depression or were initially misdiagnosed.

Supplementary Fig. 1 shows a flow chart for the study cohorts. The follow-up time was up to 18 years from the index diagnosis until death or the end of 2018, whichever came first.

### Measurements

The main outcome measures were all-cause and cause-specific mortality defined by the ICD-10 codes (Supplementary Table 1). We used all available information on causes of death, including underlying and contributory causes of death. For the main analyses, if more than one cause of death were recorded, we used the following hierarchy: suicides and events of undetermined intent; substance-use related causes (alcohol-related causes and drug-related causes); accidents (unintentional injuries by external cause); cardiovascular diseases (including ischaemic heart disease and cerebrovascular disease); cancer (malignant neoplasms); and other causes not belonging to any of the above categories. In these analyses, one patient could contribute to only one cause of death group.

In addition, for supplementary analyses, we used a different classification and identified all deaths with a specific cause of death recorded. In these models, one patient could contribute to more than one cause of death group. Alcohol-related causes of death were defined according to the Statistics Finland definition and drug-related causes of death were defined according to the European Monitoring Centre for Drugs and Drug Addiction definition.

The covariates were defined based on the information available in the registers and diagnoses defined by the ICD-10 codes (Supplementary Table 1): gender; age at index diagnosis; calendar year of index diagnosis; pre-existing psychiatric disorders defined as substance use disorders, mood disorders, anxiety disorders, personality disorders and other mental health disorders; history of intentional self-harm, including events of undetermined intent; incident (i.e. conversion to) schizoaffective disorder, bipolar disorder or schizophrenia; and somatic comorbidities, including cancer (malignant neoplasms), chronic obstructive pulmonary disease, cardiovascular diseases (ischaemic heart diseases and cerebrovascular diseases), diabetes mellitus, liver diseases and renal failure.

We used three different time periods to identify psychiatric and somatic comorbidities. For history of self-harm, we used all information available from the registers without time limits. For history of psychiatric comorbidities, we used information up to 2 years before the index diagnosis, for example to identify a pre-existing substance use disorder. For somatic comorbidities, we used information up to 2 years before the index diagnosis and also diagnoses during the follow-up, to better capture potential confounding by somatic comorbidity.

### Statistical analysis

Logistic regression models were used to estimate the difference in the distribution of patient characteristics across psychotic depression and non-psychotic depression cohorts. We used the SAS 9.4 LOGISTIC procedure to estimate the odds ratios (OR) and their 95% confidence intervals. The time to event data was analysed using the Cox proportional hazard models and the results are presented as hazard ratios (HRs) and their 95% confidence intervals. We used the PHREG procedure to estimate the HRs. The proportional hazards assumption was tested using the SAS ‘assess’ statement within the PHREG procedure, which performs graphical and quantitative methods for checking the adequacy of the model.^21^ Based on this model, the maximum absolute value from the supremum test for proportional hazards assumption was 3.5, and the test had a statistically significant *P*-value of <0.001, meaning that the proportional hazards assumption did not hold. We observed a statistically significant quantitative (non-crossover) interaction between exposure (non-psychotic depression versus psychotic depression) and time until death. Because the proportional hazards assumption was violated, we present some of the results stratified by follow-up time and our main analyses are based on the 5-year follow-up period. Causes of death were included as competing risks in the mortality models. This resulted in more conservative effect estimates compared with models where competing causes of death were not censored. All individuals remained in the at-risk population for mortality until death or end of follow-up period, whichever came first, i.e. potential subsequent change of diagnosis did not change their at-risk status.

## Results

Table 1 shows the distributions of characteristics at index diagnosis and during follow-up for 90 877 individuals diagnosed with incident severe non-psychotic depression and 19 064 individuals with incident psychotic depression. The mean follow-up time in individuals with non-psychotic depression was 8.0 years (s.d. = 5.1, median 7.7 years and interquartile range (IQR) 8.4) and in individuals with psychotic depression it was 8.7 years (s.d. = 5.3, median 8.6 years and IQR: 8.9). The follow-up time was slightly longer in the groups with psychotic depression because psychotic depression diagnoses were more common in the earlier years of the study period, i.e., before 2010.

**Table 1:** Characteristics for individuals with psychotic depression and severe non-psychotic depression^a^

Characteristic	Non-psychotic depression	Psychotic depression	OR	95% CI
Individuals, *n*	90 877	19 064	N/A	–
Follow-up time, years: mean (s.d.)	8.0 (5.1)	8.7 (5.3)	N/A	–
Gender, *n* (%)				
Female	55 900 (61.5)	10 932 (57.3)	0.83	0.80–0.86
Male	34 977 (38.5)	8132 (42.7)	1.19	1.15–1.23
Age at index diagnosis, mean (s.d.)	40.7 (13.2)	40.4 (14.1)	N/A	
Age group at index diagnosis, years: *n* (%)				
18–27	20 528 (22.6)	4938 (25.9)	1.21	1.17–1.26
28–37	16 673 (18.4)	3321 (17.4)	0.94	0.90–0.98
38–47	19 299 (21.2)	3626 (19.0)	0.87	0.83–0.90
48–57	25 397 (27.9)	4718 (24.8)	0.84	0.81–0.87
58–65	8980 (9.9)	2461 (12.9)	1.35	1.29–1.42
Year of index diagnosis, *n* (%)				
2000–2004	16 444 (18.1)	4719 (24.8)	1.48	1.43–1.54
2005–2009	25 098 (27.6)	5483 (28.8)	1.06	1.02–1.10
2010–2014	27 112 (29.8)	5179 (27.2)	0.88	0.85–0.91
2015–2018	22 223 (24.5)	3683 (19.3)	0.74	0.71–0.77
Pre-existing psychiatric disorders,^b^ *n* (%)	32 742 (36.0)	9660 (50.7)	1.83	1.77–1.89
Substance use disorder	5187 (5.7)	1663 (8.7)	1.53	1.44–1.62
Mood disorder	25 426 (28.0)	8249 (43.3)	1.97	1.91–2.04
Anxiety disorder	13 204 (14.5)	3853 (20.2)	1.50	1.44–1.56
Personality disorder	2819 (3.1)	1434 (7.5)	2.56	2.40–2.73
Other mental health disorder	3355 (3.7)	1313 (6.9)	1.94	1.81–2.07
History of intentional self-harm,^c^ *n* (%)	2779 (3.1)	1235 (6.5)	2.20	2.05–2.35
Comorbidities,^d^ *n* (%)	21 769 (23.9)	4740 (24.9)	1.05	1.01–1.09
COPD	1610 (1.8)	341 (1.8)	1.01	0.90–1.14
CVD	6672 (7.3)	1452 (7.6)	1.04	0.98–1.10
Diabetes mellitus	5244 (5.8)	1240 (6.5)	1.14	1.07–1.21
Liver diseases	2258 (2.5)	443 (2.3)	0.93	0.84–1.03
Malignant neoplasms	5648 (6.2)	1164 (6.1)	0.98	0.92–1.05
Renal failure	624 (0.7)	179 (0.9)	1.37	1.16–1.62
Transitions after index diagnosis, *n* (%)				
No state transitions^e^	77 900 (85.7)	13 864 (72.7)	0.44	0.43–0.46
Any conversion^f^	7006 (7.7)	3291 (17.3)	2.50	2.39–2.61
Died during follow-up	6490 (7.1)	2188 (11.5)	1.69	1.60–1.77
Died without conversion	5971 (6.6)	1909 (10.0)	1.80	1.70–1.90
Died after conversion	519 (0.6)	279 (1.5)	1.16	0.99–1.35

a. Individuals aged 18–65 years at index diagnosis during the years 2000–2018. Unadjusted odds ratios (OR) with 95% CI for individuals with psychotic depression compared with individuals with severe non-psychotic depression.

b. Based on the ICD-10, Finnish modification codes.

c. Including events of undetermined intent.

d. Any of the selected comorbidities (chronic obstructive pulmonary disease (COPD); cardiovascular diseases (CVDs), including ischaemic heart disease and cerebrovascular disease; diabetes mellitus; diseases of liver; malignant neoplasms; and renal failure).

e. Did not have a conversion to schizoaffective disorder, bipolar disorder or schizophrenia and did not die during follow-up.

f. Any conversion to schizoaffective disorder, bipolar disorder or schizophrenia during follow-up.

N/A, not applicable.

The proportion of men was slightly higher in the psychotic depression group than in non-psychotic depression group. The mean age at index diagnosis was about 40 years in both cohorts. Pre-existing psychiatric diagnoses were more common in the psychotic depression group compared with the non-psychotic depression group.

Individuals with psychotic depression had over twofold higher odds of having a pre-existing personality disorder than individuals with non-psychotic depression. Similarly, individuals with psychotic depression had over twofold higher odds of having a history of intentional self-harm than individuals with non-psychotic depression. No clear differences in somatic comorbidity were observed between the cohorts.

In those individuals who had a change of diagnosis, most individuals had only one change of diagnosis to the selected psychiatric disorders, i.e. conversion to schizoaffective disorder, bipolar disorder or schizophrenia during the follow-up (Supplementary Figs. 2 and 3). Individuals with psychotic depression had over a 10-fold higher probability (HR = 11.79, 95% CI 10.00–13.89) of conversion to schizoaffective disorder, about a 6-fold higher probability (HR = 6.25, 95% CI 5.59–7.00) of conversion to schizophrenia and about a 40% higher probability of conversion to bipolar disorder (HR = 1.44, 95% CI 1.37–1.51) than individuals with non-psychotic depression, when controlling for confounders (Table 2). In absolute numbers, about 17% of individuals with psychotic depression and 8% of individuals with non-psychotic depression had any conversion, and in both cohorts, the most common conversion was to bipolar disorder. About 11% of the individuals with psychotic depression and 7% with non-psychotic depression had a conversion to bipolar disorder. About 5% of the individuals with psychotic depression and 1% with non-psychotic depression had a conversion to schizophrenia.

**Table 2:** Probability of conversion to schizoaffective disorder, bipolar disorder and schizophrenia in individuals with psychotic depression compared with individuals with severe non-psychotic depression^a^

	Non-psychotic depression, *n* (%)	Psychotic depression, *n* (%)	Adjusted^b^		Fully adjusted^c^	
	(*n* = 90 877)	(*n* = 19 064)	HR	95% CI	HR	95% CI
Any conversion	7006 (7.7)	3291 (17.3)	1.92	1.86–1.98	1.80	1.74–1.86
Schizoaffective disorder	200 (0.2)	541 (2.8)	12.91	10.98–15.19	11.79	10.00–13.89
Bipolar disorder	6293 (6.9)	2023 (10.6)	1.52	1.45–1.60	1.44	1.37–1.51
Schizophrenia	629 (0.7)	906 (4.8)	6.79	6.07–7.59	6.25	5.59–7.00

a. Individuals aged 18–65 years at index diagnosis during the years 2000–2018.

b. Adjusted for gender and age at index diagnosis.

c. Adjusted for gender, age at index diagnosis, year of index diagnosis, pre-existing personality disorder, pre-existing substance use disorder and history of intentional self-harm. Death and other conversions as competing risks.

HR, hazard ratio.

No clear differences were observed in the time until conversion or the age at conversion between the two cohorts (Supplementary Table 2). In both cohorts, the median time until conversion was about 2 years after the index diagnosis, and this applied to all three conversion conditions (i.e. schizoaffective disorder, bipolar disorder and schizophrenia). In other words, if a conversion occurred, it occurred relatively shortly after the first-episode of psychotic depression and the first conversion diagnosis was stable, i.e. progression to other disorders occurred very rarely.

Within psychotic depression, conversions were associated with reduced risk of death, but within non-psychotic depression, conversion to schizophrenia was associated with an increased risk of death (Supplementary Table 3). When individuals with psychotic depression and non-psychotic depression were compared, no differences in the risk of all-cause mortality were observed in individuals who had at least one conversion during follow-up, when controlling for confounders (Supplementary Table 4). Individuals with psychotic depression had a slightly reduced risk of death because of substance use, compared with individuals with non-psychotic depression, and this was because of the lower number of deaths because of alcoholic liver disease in those with psychotic depression. Individuals with psychotic depression had an increased risk of ‘other’ causes of death, but no clear pattern was observed in this category of deaths, as it included a wide range of various causes of death.

In contrast, when comparing individuals without conversions during follow-up, individuals with psychotic depression had a higher risk of death than individuals with non-psychotic depression (Supplementary Table 5). In other words, the excess mortality in those with psychotic depression, compared with non-psychotic depression, was associated with not having a conversion during the follow-up. The highest relative risk (HR = 2.34, 95% CI 2.11–2.59) was for suicides. The median time until death was substantially lower, both in the psychotic depression and non-psychotic depression groups, in individuals who did not experience conversion, for example for all-cause mortality, the mean time until death was around 7 years in those who experienced conversion, whereas it was around 5 years in those who did not experience conversion. This difference was largest for suicides. About half of the suicides occurred within 2 years from the index diagnosis in those without a conversion, whereas about half of the suicides occurred within 5 years in those with a conversion, both in the psychotic depression and non-psychotic depression groups.

Of the individuals with psychotic depression, 11% (2188/19064) died over the 18-year follow-up and of the individuals with non-psychotic depression the figure was 7% (6490/90877) (Supplementary Table 6). The risk of death was about 40% higher in individuals with psychotic depression compared with non-psychotic depression (HR = 1.39, 95% CI 1.33–1.46) over the 18-year follow-up, when controlled for confounders. Because we observed an interaction between exposure (non-psychotic depression versus psychotic depression) and time until death, we stratified models by survival time. Supplementary Table 6 shows time stratified models for all-cause mortality by two different definitions for survival time, first, by a conditional survival time until the start of a given time interval (i.e. provided that the individual survived until the start of the interval), and then by cumulative survival time. These models show that the relative risk of death in individuals with psychotic depression was highest during the first year after the index diagnosis and then the excess risk gradually decreased but remained elevated throughout the follow-up period. These results also show that about half of the deaths occurred within the first 5 years after the index diagnosis in both cohorts (in the psychotic depression group 1199/2188 and in non-psychotic depression group 3256/6490). Furthermore, after controlling for potential confounders, individuals with psychotic depression did not have statistically significant excess mortality within 5 to 8 years after the index diagnosis compared with individuals with non-psychotic depression. Cumulative survival time showed a comparable graded decreasing relative risk over time than what was seen with the conditional survival time.

Because of the observed potential threshold effect in mortality at around 5 years after the index diagnosis and the fact that about half of the deaths occurred within 5 years from the index diagnosis, Table 3 shows the risk of 5-year cause-specific mortality. Over the 5-year follow-up time, individuals with psychotic depression had about 60% higher risk of all-cause mortality (HR = 1.59, 95% CI 1.48–1.70), over twofold higher risk of suicide (HR = 2.36; 95% CI 2.11–2.64), about 60% higher risk of death from accidents (HR = 1.63, 95% CI 1.26–2.10), and about 60% higher risk of death because of cardiovascular diseases (HR = 1.62, 95% CI 1.33–1.98) compared with individuals with non-psychotic depression, when controlling for confounders. Psychotic depression was not associated with an increased risk of death because of substance use disorders or cancer compared with non-psychotic depression.

**Table 3:** Risk of five-year cause-specific mortality in psychotic depression compared with severe non-psychotic depression^a^

	Non-psychotic depression, *n* (%) (*n* = 90 877)	Psychotic depression, *n* (%) (*n* = 19 064)	Adjusted^b^		Fully adjusted^c^	
			HR	95% CI	HR	95% CI
All-cause mortality	3256 (3.6)	1199 (6.3)	1.71	1.60–1.83	1.59	1.48–1.70
Suicide	886 (1.0)	495 (2.6)	2.60	2.33–2.90	2.36	2.11–2.64
Substance use	966 (1.1)	223 (1.2)	1.05	0.91–1.22	0.97	0.84–1.12
Accidents	216 (0.2)	85 (0.4)	1.85	1.44–2.38	1.63	1.26–2.10
Cardiovascular diseases	354 (0.4)	138 (0.7)	1.68	1.38–2.04	1.62	1.33–1.98
Cancer	521 (0.6)	124 (0.7)	1.07	0.88–1.30	1.05	0.86–1.28
Other	313 (0.3)	134 (0.7)	1.99	1.63–2.44	1.87	1.52–2.29

a. Individuals aged 18–65 years at index diagnosis during the years 2000–2018.

b. Adjusted for gender and age at index diagnosis.

c. Adjusted for gender, age at index diagnosis, year of index diagnosis, pre-existing personality disorder, pre-existing substance use disorder, history of intentional self-harm, conversion to schizoaffective disorder, bipolar disorder or schizophrenia. Causes of death defined according to the ICD-10, Finnish modification and categorised hierarchically as listed in the table.

HR, hazard ratio.

## Discussion

### Main findings

In this large population-based cohort study with up to 18 years of follow-up, 19 064 individuals diagnosed with first-episode psychotic depression were compared with 90 877 individuals with first-episode severe non-psychotic depression to examine the risk of death associated with psychotic depression, while controlling for potential confounders, including pre-existing personality disorder, pre-existing substance use disorder and conversion to schizoaffective disorder, bipolar disorder or schizophrenia. These results add to the evidence base by showing that compared with severe non-psychotic depression, the excess risk of death in psychotic depression was not explained by these psychiatric comorbidities. To our knowledge this is the first study to establish this association.

In addition, we showed that pre-existing personality disorder, substance use disorder or history of intentional self-harm, despite these being more prevalent in people with psychotic depression than in those with non-psychotic depression, did not explain the excess risk of death in individuals with psychotic depression.

Major strengths of this study were the improved precision of the effect estimates because of the relatively large number of individuals diagnosed with psychotic depression and the control for other psychiatric disorders that have been indicated as potential confounders in previous research. These findings have several important implications in terms of diagnosis, treatment and future research on psychotic depression.

### Interpretation of our findings and comparison with existing literature

Previous research has showed that comorbid personality disorder and substance use disorders are more common in those with psychotic depression than in people with non-psychotic depression,^7,20^ and that personality disorder and substance use disorders are associated with psychosis and with increased mortality.^22–24^ Our findings showed that these conditions did not explain the excess mortality in those with psychotic depression compared with severe non-psychotic depression. In other words, although these psychiatric comorbidities may add to the total burden of psychiatric morbidity, other factors are more important in determining the excess risk of mortality in psychotic depression.

During the follow-up, 17% of the individuals with psychotic depression experienced a conversion to schizoaffective disorder (3%), bipolar disorder (11%) or schizophrenia (5%). Previous studies have found comparable conversion rates in patients with psychotic depression.^16^ In absolute numbers, both in people with psychotic depression and non-psychotic depression, the most common conversion was to bipolar disorder (about 40% higher probability in those with psychotic depression than in those with non-psychotic depression), but relatively more of those with psychotic depression experienced a conversion either to schizoaffective disorder (about 12-fold higher probability in people with psychotic depression than in those with non-psychotic depression) or to schizophrenia (about 6-fold higher probability in people with psychotic depression than in those with non-psychotic depression) than in individuals with non-psychotic depression.

Conversions beyond the first conversion were very rare in this population, i.e. the initial conversions were stable over time. Bipolar disorder and schizophrenia are both associated with increased risk of death.^25,26^ However, in people with psychotic depression, any conversion, even to schizophrenia, was associated with reduced risk of death compared with those with psychotic depression who did not experience conversion, or compared with those with severe non-psychotic depression. Whereas in individuals with non-psychotic depression, conversion to schizophrenia was associated with increased mortality. Differences in the time until death, in particular for suicides, provide a potential explanation for this finding. The median time until conversion, in both individuals with psychotic depression and non-psychotic depression, was around 2 years. Also, the mean time until suicide was around 2 years, both in people with psychotic depression and with non-psychotic depression, in those who did not experience conversion. In other words, mortality was a competing event for conversion, and thus individuals with psychotic depression died more often before receiving a conversion diagnosis. Other potential explanations are that those who later experienced conversion received more effective treatment after their index diagnosis or that the total burden of their symptoms was lower, or their symptoms were qualitatively different, than in those who did not experience conversion. Future studies should aim to establish potential differences in treatment and course of symptoms in individuals with incident psychotic depression.

Of the individuals with psychotic depression, 12% died during the follow-up period. Half of the deaths occurred within 5 years from the first episode of psychotic depression. Mortality in people with psychotic depression was relatively highest during the first year after the first episode of psychotic depression, but excess mortality was observed during the full 18-year follow-up compared with people with non-psychotic depression. This finding is in line with a previous 15-year follow-up study on mortality in patients with psychotic depression.^8^ In our study, the highest relative risk in those with psychotic depression was for suicides, for which the excess mortality was twofold compared with people with non-psychotic depression. Previous meta-analyses have shown comparable relative risks for completed suicides and for suicide attempts.^9,17^

Our findings showed that the excess mortality only applied to those who did not experience conversion after the initial diagnosis of psychotic depression, when compared with those with severe non-psychotic depression. This finding suggests that there may be two subgroups within individuals with first-episode psychotic depression for whom the prognosis is very different. It would be important to identify the clinical characteristics of these individuals at the first episode of psychotic depression, so that the treatment and management of individuals with psychotic depression could be targeted appropriately and effectively. Clinicians should keep in mind that psychotic depression is associated with a substantially increased acute and long-term risk of death compared with severe non-psychotic depression. Increased risk of death because of a range of causes, including suicides, accidents and cardiovascular diseases, together with increased psychiatric comorbidity support a holistic approach to management of psychotic depression.

### Limitations

As this study was based on registers collected for administrative purposes, the study has limitations. Psychotic symptoms are known to be poorly identified in clinical contexts and thus also psychotic depression is underdiagnosed.^3,10^ Thus, our effect estimates are likely underestimates because patients with psychotic depression are, to some unknown extent, misclassified as having severe non-psychotic depression. However, it can be assumed that in a hospital context this misclassification has less impact than, for example in primary care.^27^ We excluded individuals with a history of schizophrenia spectrum disorders or bipolar disorder and, by using the 14-day exclusion period, those who likely had these conditions undiagnosed at the time of first-episode psychotic depression diagnosis. Subsequent changes in these diagnoses were adjusted for. The purpose of this design was to examine the disease process and prognosis for psychotic depression independent of these psychiatric disorders. However, it is likely that some residual confounding by these disorders remain in the models, for example because of the known delay in psychiatric diagnoses. Similarly, the at-risk status was defined at index diagnosis (start of follow-up) and any subsequent changes in diagnoses did not change the risk status. The purpose of this was to establish the prognosis of psychotic depression at the time of first-episode psychotic depression in relation to cause-specific mortality. Because duration of psychotic symptoms cannot be established using hospital discharge data, we cannot establish whether the patient had psychotic symptoms at the time of death. However, our findings show that ever experiencing psychotic depression can be used as a risk marker for increased mortality compared with severe depression without psychotic symptoms. We controlled for various potential confounders, but because the administrative registers we used do not include information on all potential confounders, such as unemployment or living alone, some residual confounding remains in the models. However, because we used severe non-psychotic depression as the control cohort, unmeasured confounding probably has less impact than in studies that have used less severe depression as the control. In addition, controlling for personality disorder and substance use disorders has controlled some of the unmeasured confounding because these covariates are associated, for example, with unemployment and living alone. Furthermore, our findings within psychotic depression are less likely affected by this potential source of bias.

### Implications

In conclusion, psychotic symptoms markedly add to the mortality risk associated with severe depression and these symptoms have an impact on both the short- and long-term prognosis of psychotic depression. Treatment and management of psychotic depression should take into account the total range and burden of psychiatric symptoms experienced by the patient, including affective and psychotic symptoms. Because psychotic symptoms are often missed in clinical contexts,^2,3^ all patients with severe depression should be actively and frequently monitored with enhanced information gathering, for example, from family members. Regular psychiatric or other healthcare contacts after diagnosis of severe depression may increase chances of identifying psychotic symptoms, such as paranoid ideas, at an early stage. These results underline the need to treat psychotic depression promptly with a combination of an antidepressant and an antipsychotic.^5,28^ Early intervention may reduce the excess mortality associated with psychotic depression, particularly as a result of suicides and other external causes.

## Data Availability

The data that support the findings of this study are available from the institutions maintaining the data.

## Supplementary material

For supplementary material accompanying this paper visit https://doi.org/10.1192/bjp.2022.140. click here to view supplementary material
