# Supplementary material for: Mortality in psychotic depression: 18-year follow-up study
Source: Br J Psychiatry. 2023 Jan;222(1):37–43. doi: 10.1192/bjp.2022.140 (PMC10895511; doi:10.1192/bjp.2022.140)
Supplement: Supplementary file 1 [file S0007125022001404sup001.docx]

**ONLINE SUPPLEMENT**

**Mortality in psychotic depression: 18-year follow-up study**

Tapio Paljärvi, Jari Tiihonen, Markku Lähteenvuo, Antti Tanskanen, Seena Fazel, Heidi Taipale

**S1 Table.** The International Classification of Diseases, tenth revision (ICD10), Finnish modification codes used in the study to define cohorts, covariates, and outcomes.

| **Description** | **ICD10 codes** |
| --- | --- |
| Malignant neoplasms (cancer) | C00 – C98 |
| Diabetes mellitus | E10, E11 |
| Organic mental disorders | F00 – F09 |
| Alcohol-related causes^a^ (mental and behavioural disorders due to use of alcohol, degeneration of nervous system due to alcohol, epileptic seizures related to alcohol, alcoholic polyneuropathy, alcoholic myopathy, alcoholic cardiomyopathy, alcoholic gastritis, alcoholic liver disease, alcohol-induced acute pancreatitis, alcohol-induced chronic pancreatitis, accidental poisoning by and exposure to alcohol | F10, G312, G4051, G621, G721, I426, K292, K70, K852, K860, X45, Y15 |
| Drug-related causes^b^ (mental and behavioural disorders due to use of opioids, mental and behavioural disorders due to use of cannabinoids, mental and behavioural disorders due to use of cocaine, mental and behavioural disorders due to use of other stimulants, including caffeine, mental and behavioural disorders due to use of hallucinogens, mental and behavioural disorders due to multiple drug use and use of other psychoactive substances, accidental poisoning by and exposure to antiepileptic, sedative-hypnotic, antiparkinsonism and psychotropic drugs, not elsewhere classified + poisoning by psychostimulants with abuse potential, accidental poisoning by and exposure to narcotics and psychodysleptics [hallucinogens], not elsewhere classified + Poisoning by narcotics and psychodysleptics [hallucinogens], intentional self-poisoning by and exposure to antiepileptic, sedative-hypnotic, antiparkinsonism and psychotropic drugs, not elsewhere classified + Poisoning by psychostimulants with abuse potential, intentional self-poisoning by and exposure to narcotics and psychodysleptics [hallucinogens], not elsewhere classified + Poisoning by narcotics and psychodysleptics [hallucinogens], poisoning by and exposure to antiepileptic, sedative-hypnotic, antiparkinsonism and psychotropic drugs, not elsewhere classified, undetermined intent + Poisoning by psychostimulants with abuse potential, poisoning by and exposure to narcotics and psychodysleptics [hallucinogens], not elsewhere classified, undetermined intent + Poisoning by narcotics and psychodysleptics [hallucinogens]) | F110-F112, F120-F122, F140-F142, F150-F152, F160-F162, F190-F199, X41+T436, X42+T400-T409, X61+T436, X62+T400-T409, Y11+T436, Y12+T400-T409 |
| Schizophrenia | F20 |
| Schizotypal disorder, persistent delusional disorders, acute and transient psychotic disorders, induced delusional disorder, other nonorganic psychotic disorders, unspecified nonorganic psychosis | F21 – F24, F28, F29 |
| Schizoaffective disorder | F25 |
| Mood (affective) disorders | F30 – F39 |

(Continued)

| **Description** | **ICD10 codes** |
| --- | --- |
| Bipolar disorder | F30, F31 |
| Severe non-psychotic depression | F32.2, F33.2 |
| Psychotic depression | F32.3, F33.3 |
| Anxiety disorders (neurotic, stress-related and somatoform disorders) | F40 – F48 |
| Personality disorders (adult) | F60, F61 |
| Intellectual disability | F70 – F79 |
| Ischaemic heart disease | I20 – I25 |
| Cerebrovascular disease | I60 – I69 |
| Other chronic obstructive pulmonary disease (COPD) | J44 |
| Diseases of liver | K70 – K77 |
| Renal failure | N17 – N19 |
| Accidents | V01 – X59 |
| Suicide | X60 – X84 |
| Events of undetermined intent | Y10 – Y34 |

^a^According to Statistics Finland definition and ICD10 codes. ^b^According to the European Monitoring Centre for Drugs and Drug Addiction definition and ICD10 codes.

Individuals identified from the nation-wide registers who did not have schizophrenia-spectrum disorders or bipolar disorder before the first diagnosis of NPD or PD, n=152801

First-episode NPD identified from the base cohort, n=116872

First-episode PD identified from the base cohort, n=35929

First episode PD during the years 2000 – 2018, n=26119

Excluded, n=9810

First-episode NPD during the years 2000 – 2018, n=104790

Excluded, n=12082

Excluded, n=7020

No conversion within 14 days from the first-episode PD, n=19099

Excluded, n=13868

No conversion within 14 days from the first-episode NPD, n=90922

Excluded, n=35

No pre-existing organic mental disorders or intellectual disability, n=19064 (analysis sample)

Excluded, n=45

No pre-existing organic mental disorders or intellectual disability, n=90877 (analysis sample)

**S1 Fig.** Flow chart of cohorts for severe non-psychotic depression (NPD) and psychotic depression (PD).

**S2 Fig.** Transitions in psychotic depression (PD) during follow-up.

PD, n=19064

No transition, n=13864

Died, n=1909

Schizoaffective disorder, n=541

Bipolar disorder, n=1979

Schizophrenia, n=771

No transition, n=721

Died, n=50

Schizophrenia, n=134
(from schizoaffective disorder, n=66; bipolar disorder, n=68)

No transition, n=1740

Died, n=171

No transition, n=389

Died, n=42

Bipolar disorder, n=44

No transition, n=126

Died, n=8 (through schizoaffective disorder, n=2; bipolar disorder, n=6

No transition, n=35

Died, n=8

Schizophrenia, n=1

No transition, n=1

NPD, n=90877

No transition, n=77900

Died, n=5971

Schizoaffective disorder, n=200

Bipolar disorder, n=6281

Schizophrenia, n=525

No transition, n=466

Died, n=59

Schizophrenia, n=104
(from schizoaffective disorder, n=32; bipolar disorder, n=72)

No transition, n=5772

Died, n=437

No transition, n=143

Died, n=13

Bipolar disorder, n=12

No transition, n=94

Died, n=10 (through schizoaffective disorder, n=2; bipolar disorder, n=8

No transition, n=12

**S3 Fig.** Transitions in severe non-psychotic depression (NPD) during follow-up.

**S2 Table.** Time to conversion from severe non-psychotic depression (NPD) and psychotic depression (PD) in those who had at least one conversion during follow-up.

|  | NPD | | | PD | | |
| --- | --- | --- | --- | --- | --- | --- |
|  | No. of Individuals, n=7006 (%) | Median time until conversion, (IQR) | Mean age at first conversion, years (SD) | No. of Individuals, n=3291 (%) | Median time until conversion, (IQR) | Mean age at first conversion, years (SD) |
| First conversion to |  |  |  |  |  |  |
| Schizoaffective disorder | 200 (2.8) | 2.3 (3.9) | 33.1 (12.2) | 541 (16.4) | 1.9 (3.6) | 34.3 (12.3) |
| Bipolar disorder | 6281 (89.6) | 1.8 (3.7) | 37.4 (12.1) | 1979 (60.1) | 2.0 (3.9) | 38.0 (13.4) |
| Schizophrenia | 525 (7.5) | 2.5 (4.4) | 31.9 (12.3) | 771 (23.4) | 2.2 (3.6) | 33.0 (12.5) |

Individuals aged 18 to 65 years at index diagnosis during the years 2000 – 2018. Interquartile range, IQR. Diagnoses defined by the International Classification for Diseases, tenth revision, Finnish modification codes.

**S3 Table.** Risk of all-cause mortality by first conversion. Pairwise comparisons by conversion within psychotic depression (PD) and severe non-psychotic depression (NPD).

|  | PD | | | | | | | |
| --- | --- | --- | --- | --- | --- | --- | --- | --- |
|  | Model 1 | | Model 2 | | Model 3 | | Model 4 | |
|  | HR | 95%CI | HR | 95%CI | HR | 95%CI | HR | 95%CI |
| No conversion | 1.00 | reference | 1.38 | 1.03, 1.85 | 1.51 | 1.29, 1.76 | 1.69 | 1.29, 2.21 |
| Schizoaffective disorder | 0.72 | 0.54, 0.97 | 1.00 | reference | 0.91 | 0.66, 1.26 | 0.82 | 0.56, 1.21 |
| Bipolar disorder | 0.66 | 0.56, 0.77 | 1.09 | 0.79, 1.51 | 1.00 | reference | 0.90 | 0.66, 1.21 |
| Schizophrenia | 0.45 | 0.45, 0.78 | 1.22 | 0.83, 1.80 | 1.11 | 0.82, 1.51 | 1.00 | reference |
|  |  |  |  |  |  |  |  |  |
|  | Model 5 | |  |  |  |  |  |  |
| No conversion | 1.00 | reference |  |  |  |  |  |  |
| Any conversion | 0.66 | 0.58, 0.74 |  |  |  |  |  |  |
|  |  |  |  |  |  |  |  |  |
|  | NPD | | | | | | | |
|  | Model 1 | | Model 2 | | Model 3 | | Model 4 | |
|  | HR | 95%CI | HR | 95%CI | HR | 95%CI | HR | 95%CI |
| No conversion | 1.00 | reference | 0.92 | 0.55, 1.52 | 1.10 | 1.00, 1.21 | 0.64 | 0.49, 0.82 |
| Schizoaffective disorder | 1.09 | 0.66, 1.81 | 1.00 | reference | 1.19 | 0.71, 2.00 | 0.69 | 0.39, 1.22 |
| Bipolar disorder | 0.91 | 0.83, 1.00 | 0.84 | 0.50, 1.40 | 1.00 | reference | 0.58 | 0.44, 0.76 |
| Schizophrenia | 1.57 | 1.21, 2.03 | 1.44 | 0.82, 2.54 | 1.72 | 1.31, 2.26 | 1.00 | reference |
|  |  |  |  |  |  |  |  |  |
|  | Model 5 | |  |  |  |  |  |  |
|  | HR | 95%CI |  |  |  |  |  |  |
| No conversion | 1.00 | reference |  |  |  |  |  |  |
| Any conversion | 0.96 | 0.88, 1.05 |  |  |  |  |  |  |

Individuals aged 18 to 65 years at the index diagnosis during the years 2000 – 2018. Models 1 to 4 show relative risks for each of the conversions when compared to different reference groups separately within PD and NPD, i.e., pairwise comparisons. Model 5 shows the relative risk for any conversion. Models adjusted for sex and age at the index diagnosis. Diagnoses defined by the International Classification for Diseases, tenth revision, Finnish modification codes.

**S4 Table.** Mortality in psychotic depression (PD) compared to severe non-psychotic depression (NPD) in those who experienced at least one conversion during the follow-up.

|  | NPD, n=7006 | | | | PD, n=3291 | | | | Adjusted^a^ | | Fully adjusted^b^ | |
| --- | --- | --- | --- | --- | --- | --- | --- | --- | --- | --- | --- | --- |
|  | No. of deaths, (%) | Median time until death, (IQR) | Mean age at death, years, (SD) | No. of men who died, (%) | No. of deaths, (%) | Median time until death, (IQR) | Mean age at death, years, (SD) | No. of men who died, (%) | HR | 95%CI | HR |  |
| All-cause mortality | 519 (7.4) | 7.5 (6.6) | 51 (12.5) | 324 (62.4) | 279 (8.5) | 7.5 (7.0) | 52 (13.9) | 155 (55.5) | 1.05 | 0.91, 1.21 | 1.01 | 0.87, 1.17 |
| Suicide | 132 (1.9) | 5.8 (6.5) | 44 (12.0) | 91 (68.9) | 69 (2.1) | 4.8 (6.0) | 45 (13.5) | 41 (59.4) | 1.04 | 0.78, 1.39 | 1.02 | 0.76, 1.36 |
| Substance use | 175 (2.5) | 7.4 (5.8) | 49 (10.8) | 130 (74.3) | 62 (1.9) | 7.0 (7.3) | 49 (11.1) | 40 (64.5) | 0.69 | 0.52, 0.93 | 0.66 | 0.49, 0.88 |
| Accidents | 96 (1.4) | 7.1 (5.6) | 48 (10.7) | 69 (71.2) | 39 (1.2) | 8.8 (8.9) | 44 (11.6) | 29 (74.4) | 0.80 | 0.55, 1.15 | 0.74 | 0.51, 1.08 |
| Cardiovascular  diseases | 96 (1.4) | 8.6 (6.2) | 57 (11.5) | 64 (66.7) | 62 (1.9) | 8.0 (7.1) | 58 (12.3) | 34 (54.8) | 1.19 | 0.86, 1.64 | 1.17 | 0.85, 1.62 |
| Cancer | 70 (1.0) | 9.8 (5.4) | 59 (7.9) | 24 (34.3) | 34 (1.0) | 8.3 (4.6) | 61 (6.9) | 12 (35.3) | 0.87 | 0.57, 1.31 | 0.89 | 0.59, 1.34 |
| Other | 62 (0.9) | 7.2 (8.0) | 55 (13.6) | 32 (51.6) | 59 (1.8) | 9.0 (7.0) | 54 (14.7) | 31 (52.5) | 1.80 | 1.26, 2.58 | 1.71 | 1.19, 2.45 |

Individuals aged 18 to 65 years at index diagnosis during the years 2000 – 2018. Hazard ratios (HR) and 95% confidence intervals (95%CI). Interquartile range, IQR. ^a^Adjusted for sex and age at index diagnosis. ^b^Adjusted for sex, age at index diagnosis, year of index diagnosis, pre-existing personality disorder, pre-existing substance use disorder, history of intentional self-harm, and any of the somatic comorbidities (chronic obstructive pulmonary disease, cardiovascular diseases, diabetes mellitus, liver diseases, malignant neoplasms, renal failure). Each cause of death defined separately using all available information from the recorded causes of death according to the International Classification of Diseases, tenth revision, Finnish modification codes. One Individual can contribute to more than one cause of death category in separate models.

**S5 Table.** Mortality in psychotic depression (PD) compared to severe non-psychotic depression (NPD) in those who did not experience conversion during the follow-up.

|  | NPD, n=83871 | | | | PD, n=15773 | | | | Adjusted^1^ | | Fully adjusted^b^ | |
| --- | --- | --- | --- | --- | --- | --- | --- | --- | --- | --- | --- | --- |
|  | No. of deaths, (%) | Median time until death, (IQR) | Mean age at death, years, (SD) | No. of men who died, (%) | No. of deaths, (%) | Median time until death, (IQR) | Mean age at death, years, (SD) | No. of men who died, (%) | HR | 95%CI | HR | 95%CI |
| All-cause mortality | 5971 (7.1) | 4.7 (6.9) | 54 (12.3) | 3614 (60.5) | 1909 (12.1) | 3.8 (7.3) | 54 (13.5) | 1080 (56.5) | 1.54 | 1.47, 1.63 | 1.48 | 1.40, 1.56 |
| Suicide | 1091 (1.3) | 1.8 (4.3) | 45 (13.0) | 694 (63.6) | 577 (3.7) | 1.3 (3.7) | 46 (13.2) | 335 (58.0) | 2.67 | 2.41, 2.95 | 2.34 | 2.11, 2.59 |
| Substance use | 2067 (2.5) | 4.3 (5.9) | 52 (11.0) | 1510 (73.0) | 471 (3.0) | 3.8 (6.1) | 50 (12.2) | 325 (69.0) | 1.11 | 1.00, 1.22 | 1.03 | 0.93, 1.14 |
| Accidents | 941 (1.1) | 4.0 (5.1) | 49 (12.1) | 638 (67.8) | 262 (1.7) | 3.6 (5.6) | 49 (13.2) | 163 (62.2) | 1.37 | 1.20, 1.58 | 1.23 | 1.07, 1.41 |
| Cardiovascular  diseases | 1275 (1.5) | 6.0 (7.6) | 59 (9.7) | 873 (68.5) | 346 (2.2) | 6.2 (8.5) | 61 (9.8) | 212 (61.3) | 1.22 | 1.08, 1.38 | 1.22 | 1.08, 1.37 |
| Cancer | 1218 (1.4) | 6.0 (7.8) | 60 (9.2) | 522 (42.9) | 277 (1.8) | 5.8 (7.6) | 61 (8.9) | 129 (46.6) | 1.03 | 0.90, 1.17 | 1.08 | 0.95, 1.23 |
| Other | 711 (0.8) | 6.1 (7.6) | 58 (12.4) | 368 (51.8) | 336 (2.1) | 5.8 (7.1) | 57 (13.1) | 166 (49.4) | 2.22 | 1.95, 2.53 | 2.16 | 1.89, 2.46 |

Individuals aged 18 to 65 years at index diagnosis during the years 2000 – 2018. Hazard ratios (HR) and 95% confidence intervals (95%CI). Interquartile range, IQR. ^a^Adjusted for sex and age at index diagnosis. ^b^Adjusted for sex, age at index diagnosis, year of index diagnosis, pre-existing personality disorder, pre-existing substance use disorder, history of intentional self-harm, and any of the somatic comorbidities (chronic obstructive pulmonary disease, cardiovascular diseases, diabetes mellitus, liver diseases, malignant neoplasms, renal failure). Each cause of death defined separately using all available information from the recorded causes of death according to the International Classification of Diseases, tenth revision, Finnish modification. One Individual can contribute to more than one cause of death category in separate models.

**S6 Table.** Risk of all-cause mortality in psychotic depression (PD) compared to severe non-psychotic depression (NPD) by survival time.

|  | NPD, n=90877 | PD, n=19064 | Adjusted^c^ | | Fully adjusted^d^ | |
| --- | --- | --- | --- | --- | --- | --- |
| Time (years) | No. of deaths, n=6490 (%) | No. of deaths, n=2188 (%) | HR | 95%CI | HR | 95%CI |
| Conditional survival time, years^a^ |  |  |  |  |  |  |
| 0 – 1 | 1001 (1.1) | 427 (2.2) | 1.96 | 1.75, 2.19 | 1.91 | 1.70, 2.14 |
| 1 – 3 | 1251 (1.4) | 447 (2.3) | 1.66 | 1.49, 1.85 | 1.53 | 1.37, 1.71 |
| 3 – 5 | 1004 (1.1) | 325 (1.7) | 1.52 | 1.34, 1.72 | 1.34 | 1.18, 1.52 |
| 5 – 8 | 1257 (1.4) | 346 (1.8) | 1.29 | 1.14, 1.45 | 1.10 | 0.98, 1.24 |
| 8 – 18 | 1977 (2.2) | 643 (3.4) | 1.28 | 1.17, 1.40 | 1.28 | 1.12, 1.34 |
| Cumulative survival time, years^b^ |  |  |  |  |  |  |
| 3 | 2252 (2.5) | 874 (4.6) | 1.79 | 1.66, 1.94 | 1.70 | 1.57, 1.84 |
| 5 | 3256 (3.6) | 1199 (6.3) | 1.71 | 1.60, 1.83 | 1.59 | 1.48, 1.70 |
| 8 | 4513 (5.0) | 1545 (8.1) | 1.59 | 1.50, 1.69 | 1.45 | 1.37, 1.54 |
| 18 | 6490 (7.1) | 2188 (11.5) | 1.45 | 1.38, 1.52 | 1.39 | 1.33, 1.46 |

Individuals aged 18 to 65 years at index diagnosis during the years 2000 – 2018. Hazard ratios (HR) and 95% confidence intervals (95%CI). ^a^Risk of death within a given time interval provided that the Individual survived until the start of the interval. Risk of death up to a given survival time, i.e., within the given years of follow-up. Cut-off values were selected based on the distribution of deaths during the follow-up period. ^c^Adjusted for sex and age at index diagnosis. ^d^Adjusted for sex, age at index diagnosis, year of index diagnosis, pre-existing personality disorder, pre-existing substance use disorder, history of intentional self-harm, and conversion to schizoaffective disorder, bipolar disorder, or schizophrenia. Causes of death defined according to the International Classification of Diseases, tenth revision, Finnish modification.
